# Supplementary material for: A Rapamycin-Based GMP-Compatible Process for the Isolation and Expansion of Regulatory T Cells for Clinical Trials
Source: Mol Ther Methods Clin Dev. 2018 Jan 31;8:198–209. doi: 10.1016/j.omtm.2018.01.006 (PMC5850906; doi:10.1016/j.omtm.2018.01.006)
Supplement: Document S1. Figure S1 and Table S1 [file mmc1.pdf]

OMTM, Volume *8*

Supplemental Information

A Rapamycin-Based GMP-Compatible Process for the Isolation and Expansion of Regulatory T Cells for Clinical Trials

Henrieta Fraser, Niloufar Safinia, Nathali Grageda, Sarah Thirkell, Katie Lowe, Laura J. Fry, Cristiano Scottá, Andrew Hope, Christopher Fisher, Rachel Hilton, David Game, Paul Harden, Andrew Bushell, Kathryn Wood, Robert I. Lechler, and Giovanna Lombardi

Supplementary Material

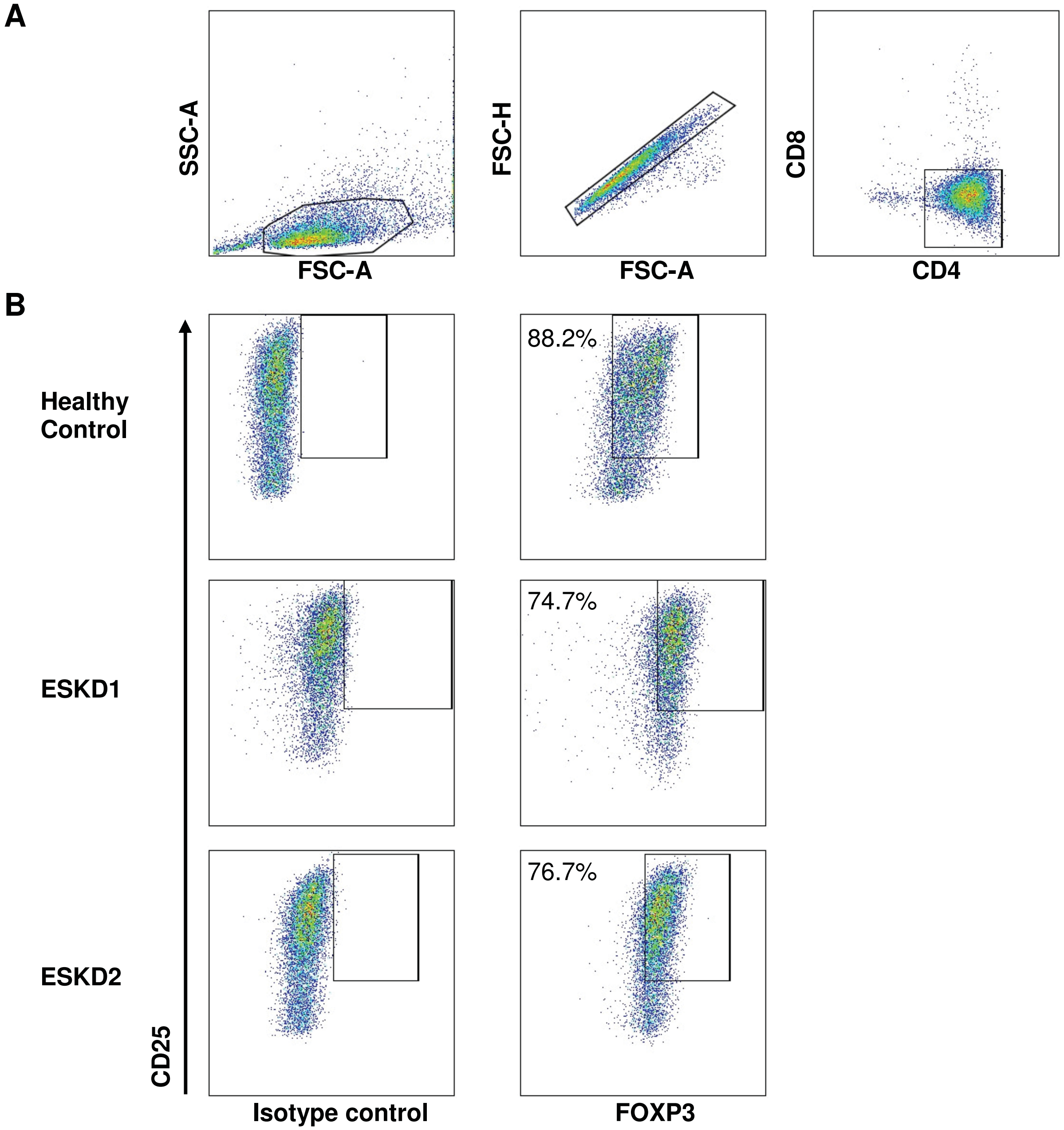

Figure S1: Gating strategy and flow cytometric staining patterns. A. Gating strategy for identification of $CD4^+CD25^+FOXP3^+$ cells. Cell doublets were excluded from the analysis on the basis of forward scatter area and height (FSC-A and FSC-H, respectively). **B.** Dot plots showing the percentage of $CD4^+CD25^+FOXP3^+$ cells for one healthy control and two patients included in the study.

Table S1: Demographics of patients included in the study.

	Gender	Ethnicity	Age	Therapeutic agents	Condition leading to ESKD (Months on dialysis prior recruitment)	Co-morbidities
**ESKD1**	Female	Caucasian	52	Venofer on dialysis<br><br>Adcal<br>Candesartan<br>Alfacalcidol	Autosomal dominant polycystic kidney disease (50)	Obesity<br>(gastric band 2011)
**ESKD2**	Female	Afro-Caribbean	49	Epo, iron and alfacalcidol on dialysis<br><br>Atorvastatin<br>Atenolol<br>Amlodipine<br>Candesartan<br>Omeprazole<br>Tramadol	Hypertension (106)	Hyperparathyroidism /parathyroidectomy 2009<br>Ischaemic heart disease
