# Supplementary material for: A Rapamycin-Based GMP-Compatible Process for the Isolation and Expansion of Regulatory T Cells for Clinical Trials
Source: Mol Ther Methods Clin Dev. 2018 Jan 31;8:198–209. doi: 10.1016/j.omtm.2018.01.006 (PMC5850906; doi:10.1016/j.omtm.2018.01.006)
Supplement: Document S2. Article plus Supplemental Information [file mmc2.pdf]

Molecular Therapy
Methods & Clinical Development
Original Article

A Rapamycin-Based GMP-Compatible Process for the Isolation and Expansion of Regulatory T Cells for Clinical Trials

Henrieta Fraser,[1,5] Niloufar Safinia,[1,5] Nathali Grageda,[1] Sarah Thirkell,[1] Katie Lowe,[1] Laura J. Fry,[1] Cristiano Scottá,[1] Andrew Hope,[1] Christopher Fisher,[1] Rachel Hilton,[2] David Game,[2] Paul Harden,[3] Andrew Bushell,[4] Kathryn Wood,[4] Robert I. Lechler,[1] and Giovanna Lombardi[1]

[1]Division of Transplantation, Immunology and Mucosal Biology, King's College London, London, UK; [2]The Department of Nephrology and Transplantation, Guy's Hospital, Guy's and St. Thomas NHS Foundation Trust; [3]Oxford Transplant Centre, Oxford, UK; [4]Transplantation Research Immunology Group, Nuffield Department of Surgical Sciences, University of Oxford, Oxford, UK

The concept of regulatory T cell (Treg)-based immunotherapy has enormous potential for facilitating tolerance in autoimmunity and transplantation. Clinical translation of Treg cell therapy requires production processes that satisfy the rigors of Good Manufacturing Practice (GMP) standards. In this regard, we report our findings on the implementation of a robust GMP compliant process for the *ex vivo* expansion of clinical grade Tregs, demonstrating the feasibility of this developed process for the manufacture of a final product for clinical application. This Treg isolation procedure ensured the selection of a pure Treg population that underwent a 300-fold expansion after 36 days of culture, while maintaining a purity of more than 75% CD4$^+$CD25$^+$FOXP3$^+$ cells and a suppressive function of above 80%. Furthermore, we report the successful cryopreservation of the final product, demonstrating the maintenance of phenotype and function. The process outlined in this manuscript has been implemented in the ONE study, a multicenter phase I/IIa clinical trial in which cellular therapy is investigated in renal transplantation.

INTRODUCTION

Naturally occurring, thymus-derived, CD4$^+$CD25$^+$FOXP3$^+$ regulatory T cells (Tregs) play a critical role in various immunological processes, favoring homeostasis. These cells are responsible for the induction and maintenance of peripheral tolerance to both self and foreign antigens.

Research in animal models has demonstrated that the adoptive transfer of murine Tregs can be used to treat several autoimmune diseases, including type I diabetes,[1] multiple sclerosis,[2] and rheumatoid arthritis,[3] in animal models. The pathophysiology of these diseases in man proposes that they have arisen, in part, due to a deficiency in Treg number and/or functional defects.[4,5] In the context of transplantation, we have demonstrated that *in vitro* expanded murine Tregs can induce indefinite heart allograft survival and skin graft prolongation,[6–9] with further studies reporting the prevention of graft-versus-host disease (GVHD) following bone marrow transplantation.[10,11]

A key breakthrough in the translational potential of Treg cell therapy was the demonstration that human Tregs could be successfully isolated and expanded *ex vivo* while maintaining immunoregulatory function. Moreover, we have also demonstrated that the adoptive transfer of polyclonally expanded human Tregs protects from alloimmune-mediated human vessel and skin pathology and induces increased survival of transplanted islets in humanized mouse models of transplantation.[12–17]

More importantly, the isolation and expansion of Good Manufacturing Practice (GMP)-compliant Tregs has enabled the application of these cells in the clinic, leading to Treg adoptive transfer in phase I clinical trials of bone marrow transplantation and type I diabetes.[18–21] Data from such trials have not only proven to be invaluable in establishing the safety and efficacy of Treg-based therapy, but has encouraged the broader application of such cell therapy, including trials in the setting of solid organ transplantation. One such trial is the recently completed ONE study (NCT02129881), a multicenter phase I/II study funded by the European Union FP7 program investigating the safety and potential efficacy of infusing *ex vivo* expanded Tregs, and other regulatory cells, in the context of kidney transplantation.

The success of a clinical trial such as the ONE study requires a highly reproducible process for the sustained manufacture of autologous patient-derived Tregs. To date, processes for the isolation of autologous Tregs have predominantly used immunomagnetic bead

Received 31 July 2017; accepted 16 January 2018;
https://doi.org/10.1016/j.omtm.2018.01.006.

[5]These authors contributed equally to this work.

Correspondence: Niloufar Safinia, Division of Transplantation, Immunology and Mucosal Biology, King's College London, London, UK.
E-mail: niloufar_safinia@yahoo.com

 © 2018 The American Society of Gene and Cell Therapy.
This is an open access article under the CC BY-NC-ND license (http://creativecommons.org/licenses/by-nc-nd/4.0/).

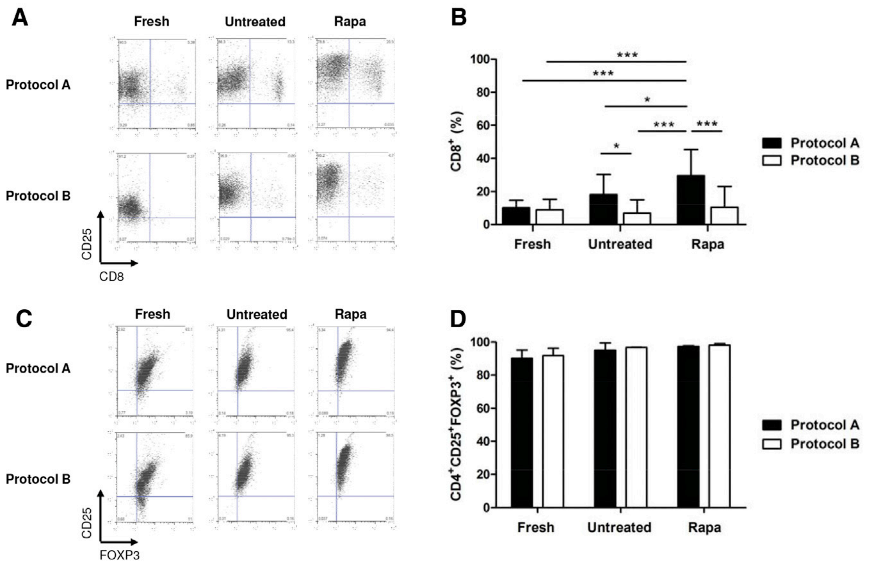

Figure 1. Comparison of Isolation Strategies, Protocol A, CD25 Enrichment, and Protocol B, CD8 Depletion and CD25 Enrichment, on the Purity of Treg Lines

(A) Representative plots depicting the percentage of contaminating CD8$^+$ cells in cultures on isolation (fresh) and after 36 days of expansion in the presence and absence of rapamycin (rapa versus untreated, respectively). (B) Influence of isolation strategy on % of CD8$^+$ cells. Protocol A shows that at final harvest (day 36), the number of CD8$^+$ cells significantly increases in both untreated and rapa-treated lines as compared to freshly isolated. Protocol B shows no difference between different treatments, and the % of CD8$^+$ cells decreases in culture in both the presence and absence of rapamycin. (C) Representative plots of % CD4$^+$CD25$^+$FOXP3$^+$ cells in cultures on isolation (fresh) and after 36 days of expansion in the presence and absence of rapamycin (rapa versus untreated, respectively). (D) There was no difference in the % of CD4$^+$CD25$^+$FOXP3$^+$ cells between protocol B or protocol A nor between cells cultured in the presence or absence of rapamycin. Data represent the average ± SD of 3 independent experiments. Statistical analysis was performed using 1-way ANOVA, and where there was a significant difference, this is indicated. *$p < 0.05$; **$p < 0.01$; ***$p < 0.001$.

isolation, offering a versatile means of cell selection in accordance with GMP processes. Despite its relative merits, the major drawback with this technique is the inability to select cells based on stricter criteria (CD25^{hi}) or multiple parameters (e.g., low expression of CD127) in contrast with fluorescence activated cell sorting (FACS), which is still not available in a closed-system GMP-compliant manner in the UK. One of the drawbacks of the bead-isolated system is that the selected Treg population may contain activated effector T cells, posing a concern in the context of subsequent expansion and clinical application, whereby the effectors may have the potential to proliferate uncontrollably and, once injected, instigate graft damage.

In order to reduce the risk that Treg preparations are contaminated with pro-inflammatory cells, many researchers have sought to establish GMP-compatible processes to improve the purity of Treg preparations for clinical application. In this regard, it has been shown that supplementing Treg cultures with the immunosuppressant rapamycin, a mechanistic target of rapamycin (mTOR) kinase inhibitor, results in the selective expansion of Tregs.[22–24]

In this study, we have established a rapamycin-based GMP-compatible process for the manufacture of GMP-compliant Tregs for cell therapy application. We have compared different reagents and conditions for the enrichment and culture of Tregs and present the validation of our process in the Biomedical Research Centre (BRC) GMP Facility at Guy's Hospital, King's College London. We demonstrated that by employing a rapamycin-based process, a phenotypically stable population of Tregs that maintain their suppressive function can be expanded and used clinically in the setting of the ONE study.

RESULTS

CD8$^+$ T Cell Depletion Is Advantageous for Obtaining a Pure and Functional Treg Population

A key component of Treg cellular therapy is the ability to isolate and expand a pure population of Tregs for clinical use. In order to develop a standardized, reproducible, isolation strategy, we compared a one-step positive selection of CD25$^+$ cells (anti-CD25-beads, Miltenyi Biotec) (protocol A) with a double-step strategy in which peripheral blood mononuclear cells (PBMCs) were first depleted of CD8$^+$ T cells (anti-CD8 beads, Miltenyi Biotec) and subsequently enriched for CD25$^+$ T cells (anti-CD25 beads, Miltenyi Biotec) (protocol B).

Freshly isolated Tregs, prepared in accordance with these two separate processes, were characterized by surface staining for CD8, CD4, CD25, and intracellular staining for the transcription factor FOXP3. It was no surprise that adopting protocol B resulted in a lower percentage of CD8$^+$ cells on isolation in comparison with protocol A (Figures 1A and 1B). However, there was no difference in the percentage of freshly isolated CD25$^+$ and FOXP3$^+$ T cells using protocol B, 91.80 ± 4.52, as compared to protocol A, 90.23 ± 4.91, $p = 0.71$ (Figures 1C and 1D).

The concept of Treg therapy is based on the expectation that the purity of the Treg population is maintained throughout *ex vivo* expansion, with no contaminants in the final Treg preparation. In order to investigate the influence of CD8 depletion and the effects of this impurity on the expanded Tregs, with respect to their expansion profile, phenotype, and suppressive function, freshly isolated Tregs from protocol A and protocol B were cultured for 36 days in X-vivo 15, supplemented with 5% human serum in the presence of interleukin-2 (IL-2) (500 IU/mL). Furthermore, and based on previous observations by our group and others, which highlight the importance of

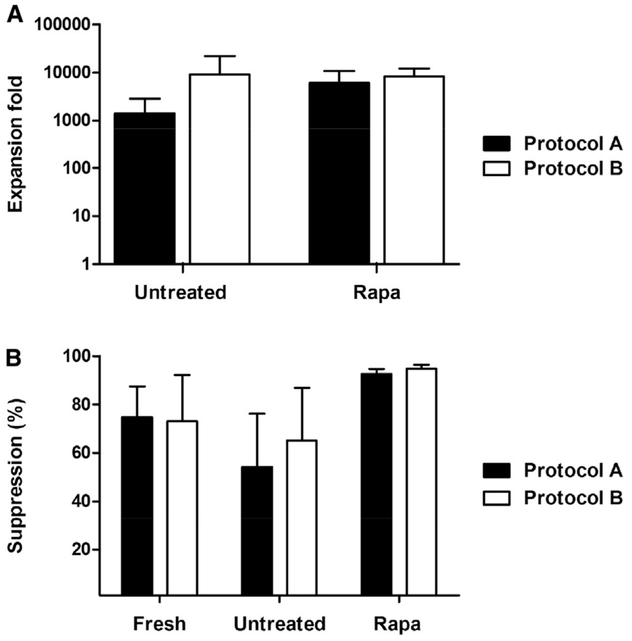

Figure 2. The Effect of Protocol A and Protocol B on the Expansion and Function of Treg Lines

(A) Tregs were expanded by stimulation with anti-CD3/CD28 beads and expanded for 36 days with IL-2 in the presence (rapa) or absence (untreated) of rapamycin. There was no difference in the fold expansion of Tregs between the different isolation strategies (protocol A and protocol B) or culture conditions. (B) The suppressive function of freshly isolated Tregs and expanded Treg lines. Suppressive ability of Tregs was measured as a decrease of proliferation of effector T cells in the presence of different concentrations of Tregs (Treg:Teff 1:1 and 1:10). Data represent the percentage Treg suppression at a Treg:Teff ratio of 1:1. Data represent mean ± SD of 3 independent experiments. Statistical analysis was performed using 1-way ANOVA and revealed no significant difference.

supplementing cultures with rapamycin,[23,24] the addition of this immunosuppressant to the cultures and the influence of this on the parameters described were also assessed.

The use of protocol A resulted in an increased percentage of CD8$^+$ T cells by final harvest, with a more pronounced increase in the rapamycin-treated cultures as compared to the untreated cultures ($p = 0.0002$), supporting previous reports of the expansion *in vivo* of murine CD8$^+$ T cells in the presence of rapamycin (Figures 1A and 1B).[25] In contrast, the use of protocol B ensured that the percentage of CD8$^+$ cells was less than 5% at the end of the 36-day expansion period (Figures 1A and 1B). However, there were no significant differences in the percentage of CD25$^+$ and FOXP3$^+$ Tregs between the two processes after expansion, and this was not influenced by the presence of rapamycin (Figures 1C and 1D). In addition, in keeping with previous studies,[26] the presence of rapamycin in the culture led to a higher expression of the CD25 molecule (Figures 1A and 1C).

Treg lines generated, using protocol A and protocol B as isolation strategies, were also assessed according to their *in vitro* expansion capacity and functional activity (Figures 2A and 2B). The addition of rapamycin to the cultures did not affect either the rate or fold of Treg expansion in either process ($p = 0.33$, Figure 2A). We next compared the regulatory function of each Treg line by measuring their ability to suppress the proliferation of co-cultured carboxyfluorescein succinimidyl ester (CFSE)-labeled T effectors stimulated with anti-CD3/CD28 beads during a 5-day culture period. Similar to the expansion profile, there was no significant difference observed between the two protocols (Figure 2B). When further characterizing the Tregs after expansion, no differences were detected between the two processes in the percentages of Tregs expressing the homing receptors CD62L, CCR4, and CLA in the presence or absence of rapamycin. However, the percentage of Tregs expressing CD27 molecules was increased when the Tregs were isolated and expanded using protocol B (data not shown). This marker on Tregs has been associated with increased Treg suppressive function.[24,27]

At this point, it was concluded that for Treg isolation, incorporation of a CD8 depletion step was advantageous over isolation based on CD25-positive selection alone. This isolation strategy reduced the presence of a potentially alloreactive CD8 population, increasing the suitability of the final product for clinical application. In addition, supplementation of rapamycin to the cultures resulted in the expansion of a pure population of Tregs with increased suppressive function. As such, the remaining experiments were conducted by adopting protocol B as isolation strategy with the addition of rapamycin to cultures, during expansion.

Depletion of CD8$^+$ T Cells and Enrichment of CD25$^+$ Cells Gives Rise to 80% of FOXP3$^+$ Cells

In order to test the efficiency of the isolation strategy and the reproducibility of the data, we generated 25 Treg lines, isolated via CD8 depletion and CD25 enrichment. The mean purity of the freshly isolated lines was 76.9% ± 12.3%, as assessed by surface staining for CD4, CD25, and intracellular staining for FOXP3 (Figure 3A). Treg immunotherapy is dependent on the expansion of a highly pure population of Tregs for cell therapy application. In this regard, the purity of the 25 lines was assessed throughout the 36 days of cultures. We show a significant increase in the purity of rapamycin-treated Tregs when compared to freshly isolated Tregs, with an average purity of Tregs cultured in the presence of rapamycin of 91.6% ± 9.3%, $p = 0.0017$ (Figure 3A). These results confirm that adopting protocol B for Treg isolation results in a consistently pure population of expanded Treg cells.

80% of the Treg Lines Expanded *In Vitro* Reached Numbers Sufficient for Clinical Application

Considering that Tregs only comprise around 1%–3% of total peripheral blood CD4$^+$ T cells in humans, and with animal studies showing that Tregs in such paucity will not suppress immune responses, we focused on the development of a strategy centered on

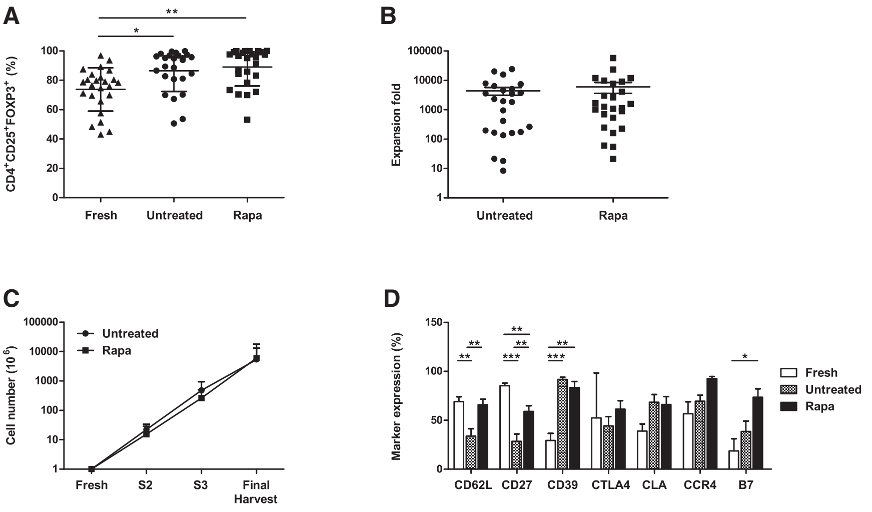

Figure 3. Purity, Expansion, and Phenotypic Characterization of Tregs on Isolation and Expansion

Tregs isolated in accordance with protocol B, stimulated with anti-CD3/CD28 beads, and expanded for 36 days with IL-2 in the presence (rapa) or absence (untreated) of rapamycin. (A) $CD4^+CD25^+FOXP3^+$ expression of freshly isolated cells and expanded Treg lines. (B) Treg fold expansion by day 36 of culture. Rapa-treated and untreated cultures exhibited similar expansion profiles. (C) Total Treg numbers post expansion. (D) Expression of regulatory markers and homing receptor expression on $CD4^+CD25^+FOXP3^+$ cells throughout culture. S- stimulation: S1, day 0; S2, day 12; S3, day 24; and final harvest, day 36. Data represent the mean ± SD of 25 independent experiments. Statistical analysis was performed using 1-way ANOVA, and where there was a significant difference, this is indicated. *p < 0.05; **p < 0.01; ***p < 0.001.

the large-scale expansion of these cells.[11] Here, we show the expansion rates for all the Treg lines at final harvest, with an average expansion rate for untreated Treg lines of 4,413 ± 1,285 and 6,059 ± 2,409 for rapamycin-treated lines (Figure 3B). In support of the robustness of the process, we clearly showed that even when starting with only 1×10^6 Tregs, and not the total number of Tregs isolated, 80% of the lines treated with rapamycin reached the maximum dose of Tregs that is planned for the ONE study (10×10^6/kg) (Figure 3C).

Treg Lines Express Markers that Characterize Functional Tregs

It is well known that Tregs are a heterogeneous population of cells expressing different surface molecules associated with their regulatory function and migratory ability. In this regard, the expression of CD62L, CD27, CD39, CTLA4, CLA, CCR4, and B7 by Tregs was assessed after 36 days of expansion (Figure 3D). Previous reports have highlighted that the expression of CD27 and CD62L correlates with high Treg suppressive ability both *in vitro* and *in vivo*.[15,16,28]

A high percentage of freshly isolated cells expressed CD62L, 69.06 ± 4.81, and CD27, 85.28 ± 2.89, which was maintained by final harvest in the presence of rapamycin, 65.9 ± 5.71, $p = 0.703$, and 59.11 ± 5.87, $p = 0.002$, respectively. However, a lower proportion of Tregs expressing these markers at final harvest was found in the untreated cultures as compared to rapamycin-treated cells, CD62L, $p = 0.001$, and CD27, $p = 0.003$, further supporting the crucial role of rapamycin in the maintenance of these markers during Treg expansion, as previously published by us.[24]

With regards to the expression of the ectonucleotidase CD39, it has been reported that the expression of CD39 on $FOXP3^+$ cells denotes a stable Treg phenotype, with the $FOXP3^+CD39^-$ Treg subset associated with the production of IL-17, although preserving their suppressive function.[29] In addition, CD39 has been described as a marker of Treg maturation and activation. In support of this, during the 36-day expansion, there was an increased percentage of cells expressing CD39 in both untreated, $p = 9.01e{-8}$, and rapamycin-treated Treg lines, $p = 0.001$, as compared to freshly isolated Tregs.

Rapamycin Ensures the Expansion of Functional and Stable Tregs, which Do Not Convert to Th17 Cells in the Presence of Pro-inflammatory Cytokines

In order to expand Tregs for clinical application, it is imperative to ensure that Tregs retain their suppressive function during *ex vivo* expansion. In this regard, following the 36-day expansion, Tregs were assessed for their suppressive capacity using a CFSE dilution assay. Figure 4A depicts a representative suppression assay, with freshly isolated Tregs before and after the 36-day expansion in the presence and absence of rapamycin. Tregs cultured in the presence of rapamycin showed an increased suppressive ability, which was also maintained at the different Treg:Teff dilutions in contrast to the untreated cultures. Interestingly, the rapamycin-treated Tregs also exhibited an increased suppressive function as compared to freshly isolated cells.

The results obtained from the 25 freshly isolated and expanded Treg lines at a 1:1 Treg:Teff ratio is represented in Figure 4B. After 36 days of culture, the percentage of suppression for the untreated Treg lines decreased to 55.2% ± 29.5% as compared to freshly isolated Tregs, 69.4% ± 19.3%, $p = 0.025$. However, Treg culture in the presence of rapamycin confirmed an increased Treg suppressive function, 80.5% ± 13.8%, as compared to untreated cultures, 55.2% ± 29.5%, $p = 0.0001$.

One of the major concerns in Treg therapy is the reported plasticity of these cells. It has been demonstrated that under inflammatory conditions, human Tregs can adopt a Th17 phenotype, conferring an undesirable pro-inflammatory phenotype and posing safety

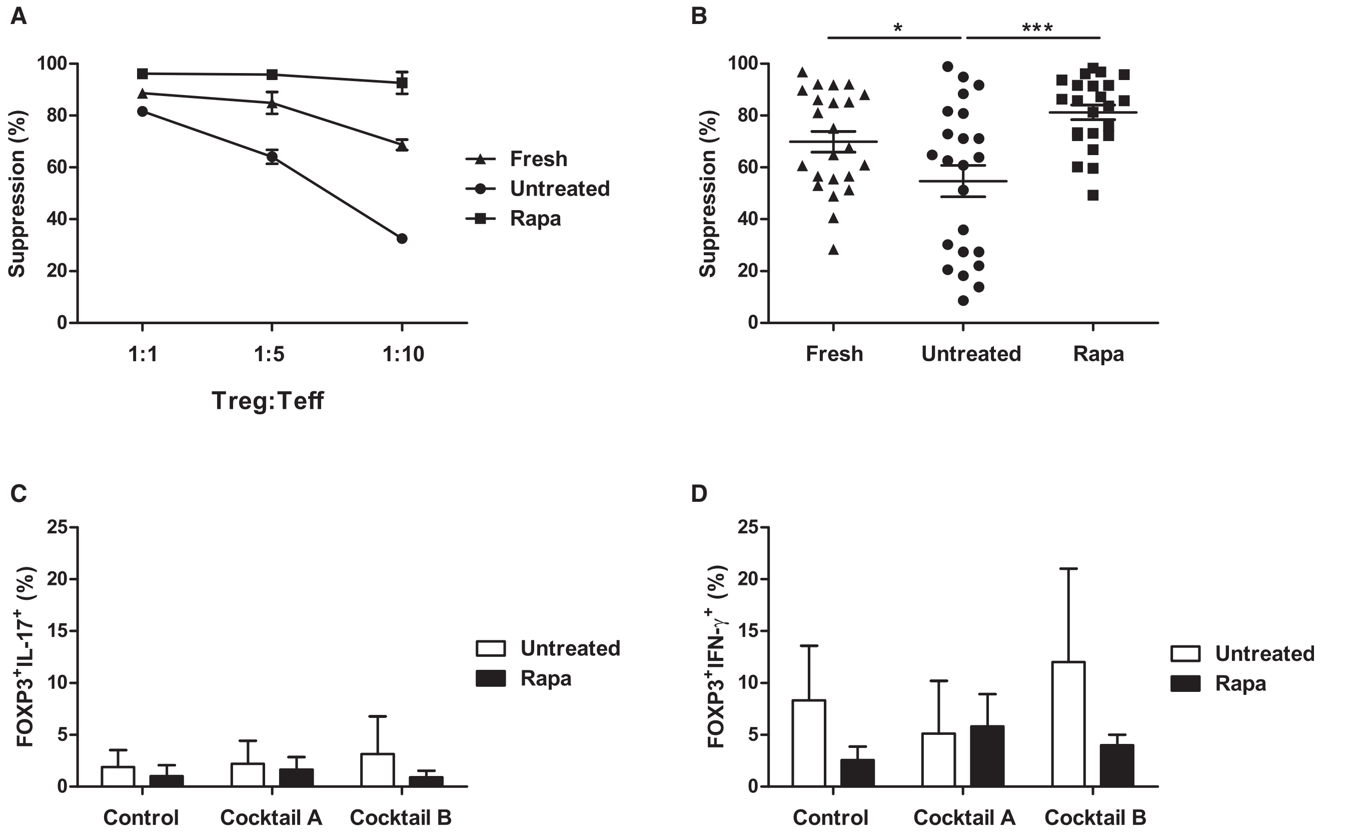

Figure 4. Suppressive Ability and Plasticity of Freshly Isolated and Expanded Treg Lines

(A) Suppressive ability of Tregs was measured as a decrease of proliferation of effector T cells in the presence of different concentrations of Tregs (Treg:Teff 1:1, 1:5, and 1:10). Graph denotes a representative suppression assay with freshly isolated Tregs before and after the 36-day expansion in the presence and absence of rapamycin. (B) Cumulative data on the average suppressive ability of 25 Treg lines following isolation (fresh) and post expansion in the presence and absence of rapamycin at a Treg:Teff ratio of 1:1. *$p < 0.05$; **$p < 0.01$; ***$p < 0.001$. Analysis of the plasticity and stability of the untreated or rapa-treated Treg lines after a 1-week culture in the presence of a pro-inflammatory milieu. (C and D) Percentage of $FOXP3^{+}IL\text{-}17^{+}$ cells (C) and $FOXP3^{+}IFN\text{-}\gamma^{+}$ cells (D) analyzed by intracellular staining. Data represent the mean ± SD of 3 independent experiments. Statistical analysis was performed using 1-way ANOVA, and where there was a significant difference, this is indicated. Control: IL-2 (10 IU/mL) only; Cocktail A: IL-2, IL-1β, IL-6, and TGF-β; Cocktail B: IL-2, IL-21, IL-23, and TGF-β (see text for details).

concerns when used as a cell therapy product.[30] To address this, at final harvest, Treg lines were stimulated with anti-CD3/CD28 beads and cultured in the presence of IL-17-inducing cytokines. Two cytokine cocktails were selected: cocktail A, IL-1β, IL-2, IL-6, and tumor growth factor beta (TGF-β); and cocktail B, IL-2, IL-21, IL-23, and TGF-β. After 5 days of culture, the percentage of $FOXP3^{+}IL\text{-}17^{+}$ and $FOXP3^{+}IFN\text{-}\gamma^{+}$ (interferon-gamma^{+}) cells was determined (Figures 4C and 4D). In comparison with untreated cultures, expansion in the presence of rapamycin resulted in a decreased frequency of $FOXP3^{+}IL\text{-}17^{+}$ (cocktail A: untreated, 2.22% ± 1.27%, and rapamycin treated, 1.65% ± 0.70%; cocktail B: untreated, 3.16% ± 2.09%, and rapamycin treated, 0.91% ± 0.36%) (Figures 4C and 4D). These results further confirm our previously published work.[24]

Generation of Treg Lines for Clinical Use

Based on the requirement for GMP-compliant production of advanced therapy medicinal products (ATMPs), necessitating reagents and consumables to be made in sterile environments, we next sought to compare the effects of the different reagents and of their combinations (Table 1) on Treg expansion profile, function, and stability during expansion. The use of GMP-compliant materials did not alter the growth or characteristics of Tregs presented so far using research-grade reagents (data not shown) and, therefore, the components included in Table 1 (and marked with asterisks) were used for all future GMP production.

As such, Figure 5 summarizes the optimal process developed, translating our preclinical work into a finely tuned GMP-compatible expansion process. In brief, Tregs were stimulated with anti-CD3/CD28 beads in a ratio of 4:1 (ExpAct Treg kit, Miltenyi Biotec) and cultured in TexMACS GMP media (Miltenyi Biotec) supplemented with 5% human serum (Lonza/Seralab). Rapamycin (Pfizer) (100 nM) was added at the beginning of the culture, whereas IL-2 (Novartis) (500 IU/mL) was added after 4 days. Both rapamycin

Table 1. Optimization of Treg Expansion

Process	Tested Reagent	Specification of Tested Reagent	Titration	Kinetics
Expansion	Cell concentration		$3x10^6$	
			$1x10^6$	
			$0.5x10^{6}$*	
	anti-CD3/ CD28 beads	Invitrogen	1:2	
			1:1	
			2:1	
			4:1	
		Miltenyi Biotec	2:1	
			4:1*	
	IL-2	Proleukin - Novartis	500 IU/ml*	day 0
				day 4*
				day 6
			1000 IU/ml	
	Rapamycin	Rapamune - Pfizer	1nM	
			10nM	
			50nM	
			100nM*	
	Medium	Lonza - Xvivo 15 w phenol red		
		Lonza - Xvivo 15 w/o phenol red		
		Invitrogen - Optimizer		
		Miltenyi Biotec - TexMACS*		
	hAB serum	Biosera - research grade	2%	
			5%	
		Lonza - male hAB - CE marked	5%*	
		Lonza – mixed hAB	5%	
		HSA	5%	
	Expansion device	Plate		
		Flask		
		Expansion bags - Miltenyi Biotec*		
		Bioreactor - Wilson Wolf		

Asterisks (*) highlight the reagents used. Different combinations of reagents, in addition to varying titrations and kinetics, were used to devise a protocol for the optimal expansion of Treg lines. Factors directly assessed consisted of Treg seeding concentrations, bead:cell ratios (alongside comparison of anti-CD3- anti-CD28-coated beads provided by different manufacturers), concentrations and timing of IL-2 supplementation, rapamycin concentrations, media from alternative manufacturers supplemented with various percentages of human serum or human serum albumin, expansion devices, and cryopreservation media and vessels.

and IL-2 were replenished every 2 to 3 days, and cells rested for 4 days prior to restimulation. Cells were restimulated every 12 days by adding activation beads, rapamycin, and IL-2. Phenotypic and functional characterization of the Tregs was carried out following final harvest at day 36.

Ex Vivo-Expanded Tregs from Patients Yield an Enriched Population, which Is Functionally Suppressive, Achieving the Release Criteria Needed for Their Clinical Application

Having developed a clinically applicable GMP process for the isolation and expansion of a pure and stable population of Tregs in the laboratory, it was of importance to validate our process in the BRC GMP Facility at Guy's Hospital prior to the clinical application of Tregs. Furthermore, in a previous publication,[31] we presented an in-depth characterization of Tregs isolated from patients with end-stage kidney disease (ESKD), concluding that patients with ESKD have similar numbers of Tregs as compared to the healthy donors. Here, we sought to isolate Tregs using the CliniMACS Plus System and compared the recovery of the isolated cells between the two patients with ESKD and a healthy donor. Despite an initial lower recovery of isolated Tregs from patients, 1×10^6 and 0.46×10^6, in comparison with the healthy donor, 5×10^6, partly explained by the amount of the starting material used, the expansion profile was comparable with Tregs expanding to numbers suitable for their clinical application (Figure 6A).

Characterization of the expanded Tregs was performed to ensure that the final product satisfied the specified release criteria in order to allow their future clinical application. Flow cytometric analysis of the Tregs at final harvest established that the percentage of $CD4^+CD25^+FOXP3^+$ cells was 88.2% for the healthy donor and 74.7% and 76.7% for the patients (Figure S1). All final products had a viability of >95% and exhibited a potent suppressor function of >80% in the classical suppression assay (Figure 6B). In addition, contamination with $CD8^+$ cells was minimal (<10% of $CD8^+$ cells in both groups) and all final products passed the necessary safety tests as defined in the release criteria (Table 2).

Cryopreservation of Expanded Tregs

In order to allow infusion of the cell product 5 days post-transplant, the feasibility of Treg cryopreservation was tested. Cryopreservation of Tregs allows flexibility in administration to assist with clinical pathways and facilitates completion of all required release assays. In addition, it allows time for transportation to different transplant centers, which would be important for ATMP trials involving centralized manufacturing sites, such as Guy's Hospital (London). For other trials, cryopreservation could also allow for repeated dosing following the production of a single Treg batch. Current experience with Treg cryopreservation is limited. Published studies differ in freeze/thaw techniques, either storing the isolated Tregs after leukapheresis (weeks or months before transplantation[32]) or, as in the clinical trial of Tregs isolated from umbilical cord blood (UCB), storing the cells after *ex vivo* expansion.[18]

Choice of an appropriate process for cryopreservation of Tregs plays a critical role in achieving a high recovery of fully functional Tregs after cryopreservation. Various different factors have been studied, leading to improvements in cryopreservation technique.[33–37] The method outlined in this manuscript resulted in a viability of >75% for both the patient Treg cultures and healthy controls when cells were thawed 12 weeks after cryopreservation. In addition, total cell recovery was

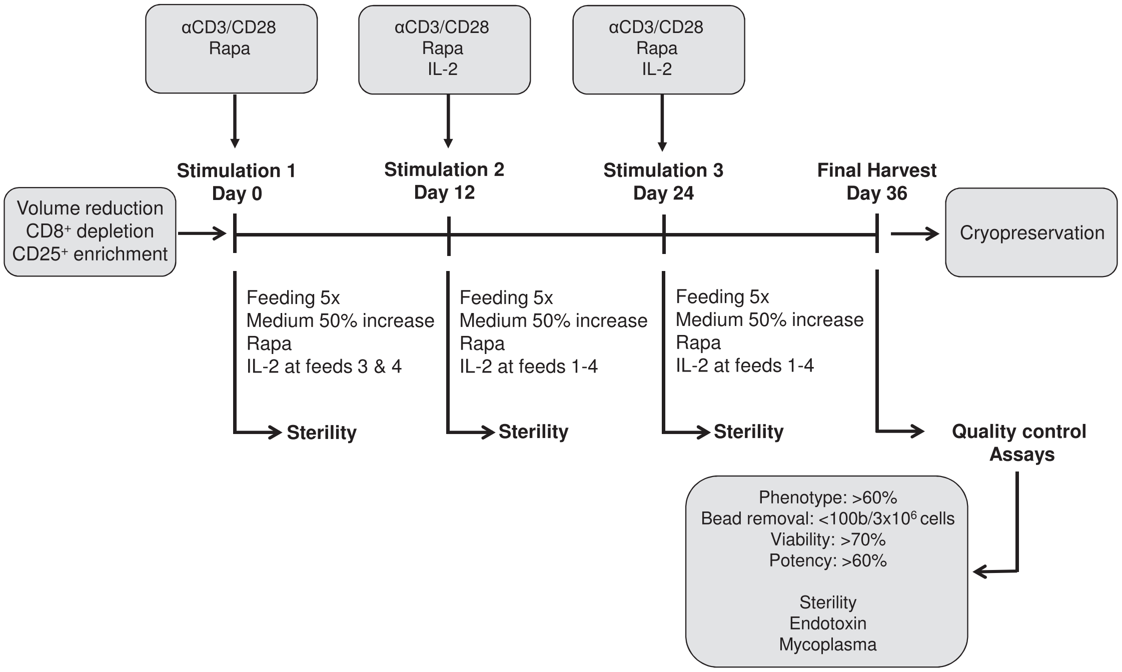

Figure 5. Schematic Representation of the GMP-Compliant Protocol for the Isolation and Expansion of Tregs for Clinical Application

Blood is volume reduced using the Sepax 2 device (Biosafe) prior to Treg isolation. $CD4^+CD25^+$ T cells are isolated using a combination of $CD8^+$ depletion (CD8 reagent, Miltenyi Biotec) and enrichment step for $CD25^+$ cells (CD25 reagent, Miltenyi Biotec) using the automated CliniMACS Plus System (Miltenyi Biotec). All processing steps are performed in closed systems. Tregs are stimulated with anti-CD3/CD28 beads in a 4:1 ratio (ExpAct Treg kit, Miltenyi Biotec) and cultured in TexMACS GMP media (Miltenyi Biotec) supplemented with 5% human serum (Lonza/Seralab). Rapamycin (Pfizer) (100 nM) is added at the beginning of the culture, whereas IL-2 (Novartis) (500 IU/mL) is added after 4 days. Both rapamycin and IL-2 are replenished every 2 to 3 days, and cells are rested for 4 days prior to restimulation. Cells are restimulated every 12 days by adding new activation beads, rapamycin, and IL-2. Phenotypic and functional characterization of the Tregs is carried out following final harvest at day 36 to ensure all final products meet the specified release criteria prior to cryopreservation and subsequent clinical application.

more than 90% for both groups. Purity, assessed by intracellular staining of FOXP3 as well as the surface markers CD4 and CD25, averaged >70% for both groups, meeting the release criteria. Importantly, the thawed cells maintained their suppressive function, with >80% suppressive capability of Tregs from patients and healthy donors (Table 2). These data show great promise for the clinical application of cryopreserved Tregs.

DISCUSSION

Clinical trials to date have confirmed the safety and hinted at the efficacy of Treg cell therapy in the context of bone marrow transplantation[18,19,21] and type I diabetes.[20] Based on data from such studies, there is a growing consensus that the therapeutic delivery of Tregs has great promise for immunosuppressive drug minimization in the field of transplantation, with a component of the ONE study specifically designed to investigate this rationale.

For Treg cell therapy to be a viable therapeutic option, the use of tailor-made GMP-compliant isolation and expansion procedures is an essential prerequisite. Organ transplant recipients demand a high purity of Tregs because infusion of non-regulatory cells into these patients may have the potential to intensify the disease process and/or lead to graft damage. Therefore, it was important for us to monitor the dynamics of Tregs throughout the isolation and expansion process, ensuring that a phenotypically and functional stable population of cells was manufactured for cell therapy application.

To this end, we sought to develop a protocol that ensured the efficient and reliable enrichment of functional human $CD4^+CD25^+$ T cells from healthy controls and patients with ESKD. The protocol that was developed (B) has now been applied in the BRC GMP facility for the isolation and expansion of GMP-compliant Tregs from patients with ESKD, and Tregs manufactured according to this process have been administered to twelve patients as part of the ONE study (NCT02129881). Following infusion, there have been no reports of toxicity or adverse events either immediately or months after Treg administration, up to a dose of 10×10^6 Tregs/kg (personal communication).

In this manuscript, we have described the fundamental principles required in the development of a Treg manufacturing process and present our final, reproducible strategy for the *ex vivo* expansion of autologous patient-derived Tregs for clinical use.

Two different isolation strategies were directly compared, protocol A and protocol B, concluding that an extra CD8 depletion step was imperative in ensuring the isolation of a pure population of Tregs.

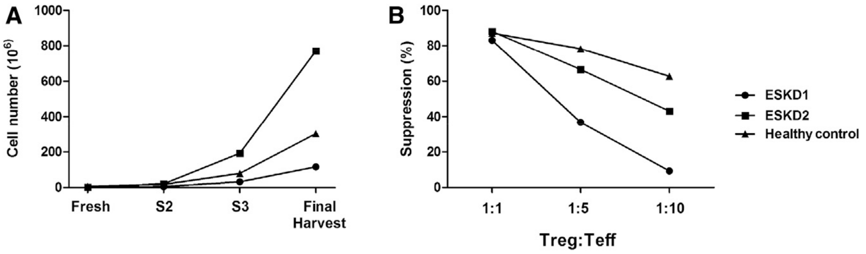

Figure 6. Expansion of Clinical Grade Tregs in the BRC GMP Facility

Tregs isolated in accordance with GMP protocols from two end-stage kidney disease patients and a healthy control. (A) Comparable final harvest cell numbers were observed between ESKD1 (117×10^6), ESKD2 (770×10^6), and the healthy control (303×10^6). (B) Suppressive ability of Tregs was measured as a decrease of proliferation of effector T cells in the presence of different concentrations of Tregs (Treg:Teff 1:1, 1:5, and 1:10). Graph denotes a suppression assay, with expanded Tregs after the 36-day expansion.

IFN-γ producing $CD8^+$ T cells have been shown to indicate a poor outcome post-transplant; thus, these cells have to be excluded from the initial isolated population in order to prevent their expansion in culture. Protocol B ensured that the percentage of $CD8^+$ T cells was kept <5% at the end of the 36-day culture period, highlighting its comparative merit.

Initially, Treg expansion processes were based purely on stimulation with anti-CD3/anti-CD28 beads and concurrent supplementation with recombinant IL-2.[21] However, improvements in this process have seen the use of rapamycin, shown to preferentially inhibit the proliferation and function of $CD25^-$ conventional effector T cells, thus permitting the preferential expansion of Tregs.[23,38,39] In accordance with this work, there is now a wealth of data from *in vitro* and *in vivo* studies favoring the use of rapamycin for Treg induction, expansion, and function.[23,38–41]

We further demonstrated that the use of rapamycin throughout the culture was critical for the *ex vivo* selective expansion of a pure (>75% $FOXP3^+$) and highly suppressive (>80% at 1:1 ratio) population of Tregs. Despite this promise, it has been reported that the addition of rapamycin to Treg cultures diminishes overall Treg expansion,[23] which may require the extension of culture periods in order to achieve therapeutic numbers. However, this solution poses a challenge in itself, bearing in mind studies reporting a loss of FOXP3 expression in human Tregs following prolonged culture.[42] In this regard, we next compared the expansion profiles of untreated and rapamycin-treated Treg lines throughout the 3 rounds of stimulations. In agreement with previous reports,[24,43] at the first round of stimulation, it was shown that the presence of rapamycin decreased the expansion rate of Tregs as compared to the untreated cultures (Figure 3C); however, this difference decreased with subsequent rounds of stimulation. As such, the data concluded that by final harvest, the use of rapamycin did not alter the expansion profile of the Treg cultures as compared to the untreated cultures, with 80% of the lines reaching numbers suitable for clinical application.

Furthermore, in keeping with previous studies,[26] the presence of rapamycin in culture led to a higher expression of CD25 molecules on Tregs while maintaining FOXP3 expression throughout the 36-day culture. Zeiser et al.[39] provided an explanation for this finding, postulating that inhibition of the mTOR pathway in the presence of IL-2 allows Tregs to be constantly activated through the STAT-5 pathways, promoting their preferential expansion and preserved FOXP3 expression. In accordance with these findings, we have incorporated more stringent release criteria to encompass FOXP3 expression. As such, the final product needs to satisfy the release criteria, with $\geq$60% of $CD4^+CD25^+FOXP3^+$ cells.

One of the principles during Treg *ex vivo* expansion is to prevent the induction and expansion of IL-17-producing cells. These inflammatory cells have the potential to arise from T effectors contaminating the Treg preparations[44] and/or $FOXP3^+$ Tregs converting to cells producing pro-inflammatory cytokines.[28] One major risk for Treg therapy is that the cells may acquire effector functions and lose their suppressive ability during inflammatory responses *in vivo*. To address this issue, Treg lines were cultured in the presence of pro-inflammatory cytokines previously reported to favor Th17 conversion.[28,45] It was concluded that rapamycin decreased the percentage of $FOXP3^+$ $IL\text{-}17^+$, paralleled by a decrease in $FOXP3^+IFN\text{-}\gamma^+$ cells, as compared to untreated Treg lines (Figures 4C and 4D). This is consistent with previous work by our group and others, demonstrating the inhibitory effects of rapamycin on Th17 cells both *in vitro* and *in vivo*.[24,46]

The next stage in the development of the GMP-compliant cell product involved the scale up process using 200 mL of blood from patients with ESKD. The CliniMACS Plus system (Miltenyi Biotec) provided a relatively versatile method for the GMP cell isolation, in which the cells were separated on a clinical level in a closed and sterile system.

We have shown that Tregs from both patients and healthy donors can be expanded after isolation using the CliniMACS System to numbers needed for the maximum dose planned for the ONE study (10×10^6/kg). Furthermore, the cells maintained their phenotype and function throughout the 36-day culture period and fulfilled the release criteria set for our clinical trials. As part of the ONE study, we have further demonstrated the consistent manufacture of the final product, reaching numbers necessary for the planned doses, allowing for the completion of the clinical trial.

Additionally, to improve logistics and allow infusion 5 days after kidney transplantation, the data presented here also conclude that, following the freeze/thaw process, Treg viability and suppressive

Table 2. Characterization of GMP-Expanded Tregs before and after Cryopreservation

	Recipient Blood	Healthy Control		ESKD1		ESKD2	
Test	specification	final harvest	defrosting	final harvest	defrosting	final harvest	defrosting
Identity	positive for CD4, CD25, and FOXP3	yes	yes	yes	yes	yes	yes
Purity	$\geq$60% of entire cell population CD4$^+$CD25$^+$FOXP3$^+$	88.2	71.7	74.7	70.5	76.7	88.6
Impurity	$\leq$10% CD8$^+$	0.41	4.68	1.82	9.61	1.6	6.35
Viability	$\geq$70% viability	96.5	76	96	93	95	82
Potency	$\geq$60% suppression	81	97	83.1	84	91.3	95

Characterization of the expanded Tregs was performed to ensure the final product satisfied the specified release criteria in order to allow their future clinical application. Release criteria: (1) positive for CD4$^+$CD25$^+$FOXP3$^+$; (2) CD4$^+$CD25$^+$FOXP3$^+$ cells $\geq$60% of live cells; (3) CD8$^+$ cells $\leq$10%; (4) viability $\geq$70%; and (5) suppression $\geq$60%. The two ESKD patients and the healthy control met the specified release criteria before and after 12 weeks of cryopreservation in vapor phase liquid nitrogen.

function was maintained. Furthermore, cryopreserving the final product allows more flexibility around the timing of the infusion and offers the possibility of administering multiple infusions in future trials. However, current knowledge of how the process of cryopreservation may affect Tregs is still limited. The data summarized in this manuscript focuses on our findings 12 weeks after Treg cryopreservation, assessing the effects of the freeze/thaw process on the expanded cells, with regard to their biology and function.

Growing interest in Tregs and enthusiasm for their potential clinical applications have intensified over recent years based on encouraging results laid down by early clinical trials. However, data concerning the efficacy of Treg cell therapy to date are limited. It is postulated that in order to achieve maximal efficacy, billions of expanded Tregs administered at multiple time points may be required. This possibility poses further challenges for the preservation of a pure population of Tregs following large-scale manufacture. Incorporation of new GMP isolation techniques, such as a GMP-compatible FACS cell sorting that is still under validation in Europe, offers the prospect of an attractive solution to address this issue, paving the way for a bright future for adoptive Treg immunotherapy.

MATERIALS AND METHODS

Cell Sources and Treg Separation and Expansion in the Research Laboratory

In the laboratory, PBMCs from healthy donors were obtained from anonymized human leukocyte cones supplied by the National Blood Transfusion Service (NHS Blood and Transplantation, NHSBT, Tooting, London, UK). Human studies were conducted in accordance with the Declaration of Helsinki and approved by the Institutional Review Board of Guy's Hospital (reference 09/H0707/86 and 13/SC/0568). PBMCs were isolated by lymphocyte (PAA, Pasching, Austria) density gradient centrifugation. CD4$^+$CD25$^+$T cells were separated using either a single-step strategy involving CD25$^+$ T cell enrichment (protocol A) or a double-step procedure consisting of a CD8$^+$ T cell depletion step followed by enrichment of CD25$^+$ T cells (protocol B). All reagents and consumables used were of clinical grade, including the magnetic-activated cell sorting (MACS) microbeads (CD8 reagent and CD25 reagent, Miltenyi Biotec, Woking, UK) used in both processes.

For development of the GMP-compliant Treg expansion process in the research laboratory, human CD4$^+$CD25$^+$ T cells were plated at 1 $\times$ 10^6/mL in culture media X-vivo 15, with or without phenol red (Lonza, Basel, Switzerland) OptMiser (Invitrogen, Paisley, UK) or TexMACS GMP medium (Miltenyi Biotech, Germany), supplemented with 2%–5% human AB serum (HS) (Seralab, Ringer, UK; Lonza, UK) or human serum albumin (Biotest, UK) containing rapamycin (1–100 nM) (Rapamune, Wyeth/Pfizer, USA). Cells were activated with Dynabeads CD3/CD28 CTS (Invitrogen, Paisley, UK) or ExpAct Treg kit (Miltenyi Biotech, Germany) at defined bead:cell ratios. IL-2 (500–1,000 IU/mL, Proleukin, Novartis, Frimley, UK) was added at day 0–4 post-activation and replenished every 2 days. Cells were restimulated every 10–12 days, with or without removing the activation beads, adding fresh beads, rapamycin, and IL-2. Expanded cells were used for further analysis at each restimulation until day 36 of expansion.

Analysis of Cell Surface and Intracellular Markers to Confirm the Identity of Freshly Isolated and Expanded Tregs

The following anti-human monoclonal antibodies (mAbs) were used for flow cytometry analysis: CD4-PerCP/Cy5.5 (clone OKT-4, eBioscience); CD25-PE (clone 4E3; Miltenyi Biotec, Woking, UK); CD62L-FITC (clone Greg-56; Invitrogen, Paisley, UK); CD8-APC (clone RPA-T8), CD39-FITC (clone eBioA1), IFN-γ-fluorescein isothiocyanate (FITC) (clone 4S.B3), IL17-PE (clone eBio64DEC17), and FOXP3-FITC (clone PCH101), all from eBioscience (San Diego, CA, USA); and CD27-PE (clone M-T271; BD Bioscience, Oxford, UK). Prior to use, all mABs were titrated using normal resting or activated PBMCs to establish optimal staining dilutions.

After harvest, cells were washed and stained with the above listed mAbs for 20 min at 4°C. Intracellular staining for FOXP3 was performed in accordance with the manufacturer's protocol (eBioscience). Appropriate isotype controls and fluorescence minus one controls were used to assign gates and flow cytometry carried out on the BD FACSCanto cell analyzer (BD Bioscience), and FlowJo software used for subsequent data analysis.

Separation of Responder T Cells and Suppression Assays

Responder CD4$^+$CD25$^-$ T cells were obtained from PBMCs by either (1) negative selection using unconjugated anti-CD8, anti-CD33 (both

Caltag, CA, USA), anti-CD14, anti-CD16, anti-CD19, anti-CD56 (all Diaclone, Gen-probe, San Diego, USA), anti-γδ T cell receptor (TCR) (clone B1.1), and glycophorin A (clone HIR-2) antibodies with pan-immunoglobulin G (IgG) microbeads and anti-CD25 microbeads (both Invitrogen, Paisley, UK) or (2) using the negative fraction obtained from the miniMACS $CD4^+CD25^+$ T Regulatory Cell Isolation Kit (Miltenyi Biotec, UK) as previously described.[24] Aliquots of the $CD4^+CD25^-$ cells were cryopreserved and used as responder cells in suppression assays.

Cryopreserved responder $CD4^+CD25^-$ T cells (Teff) were thawed and labeled with 2.5 μM CFSE (Molecular Probes, Carlsbad, CA, USA). 1×10^5/well of responder T cells were co-cultured with Tregs at different ratios (Treg:Teff 1:1, 1:5, and 1:10) in X-vivo 15 medium supplemented with 5% HS and activated by anti-CD3/CD28-coated beads (Invitrogen, Paisley, UK) in U-bottom 96-well plates. Cells were incubated at 37°C, 5% CO_2, for 5 days. After harvest, proliferation of CFSE-labeled responder cells was acquired by flow cytometry (FACSCalibur or LSRFortessa cell analyzer [BD Bioscience]) and analyzed with FlowJo software (Tree Star, OR, USA). The suppressive ability of Treg lines was assessed as the percentage decrease of Teff proliferation in the presence of Tregs. The calculation was based on the proliferation of responder T cells alone compared with the proliferation of cultures also containing Treg cells.

Treg Culture in the Presence of Pro-inflammatory Cytokines

Freshly isolated, untreated, and rapamycin-treated $CD4^+CD25^+$ T cells (5×10^5) were activated with anti-CD3- and anti-CD28-coated beads at a 1:1 bead:cell ratio and cultured for 5 days in the presence of pro-inflammatory cytokine cocktails. Cocktail A: IL-2 (10 IU/mL), IL-1β (10 ng/mL, R&D Systems, Minneapolis, MN, USA), IL-6 (4 ng/mL, R&D Systems, Minneapolis, MN, USA), and TGF-β (5 ng/mL, R&D Systems, Minneapolis, MN, USA). Cocktail B: IL-2 (10 IU/mL), IL-21 (25 ng/mL, Cell Sciences, Canton, MA, USA), IL-23 (25 ng/mL, R&D Systems, Minneapolis, MN, USA), and TGF-β (5 ng/mL, R&D Systems, Minneapolis, MN, USA). Cells cultured in complete medium supplemented with IL-2 (10 IU/mL) were used as controls to ensure their survival throughout the 5-day stability assay. At the end of the culture, cells were harvested, activation beads were removed by magnetic adherence, and cells were activated with phorbol myristate acetate (PMA) (5 ng/mL, Sigma-Aldrich, St. Louis, MO, USA), ionomycin (1 μg/mL, Sigma-Aldrich, St. Louis, MO, USA), and monensin (2 μM, eBioscience, San Diego, CA, USA) for 4 hr. Subsequently, cells were analyzed for IL-17 and IFN-γ expression by intracellular staining.

GMP-Compliant Treg Isolation, Expansion, and Cryopreservation in the BRC GMP Facility

Isolation

For the GMP validation of the process, a buffy coat was obtained from NHSBT and used as a healthy donor, whereas 200 mL of blood was obtained from two patients with ESKD on hemodialysis (inclusion/exclusion criteria as per the ONE study, NCT02129881). Patient demographics are outlined in Table S1. Informed consent was obtained from all donors prior to enrolment into the study.

Blood volume was reduced using the Sepax 2 device (Biosafe) prior to Treg isolation. $CD4^+CD25^+$ T cells were isolated using a combination of $CD8^+$ depletion (CD8 reagent, Miltenyi Biotec) and enrichment step for $CD25^+$ cells (CD25 reagent, Miltenyi Biotec) using the automated CliniMACS Plus System (Miltenyi Biotec) in the BRC GMP Facility at Guy's Hospital. All processing steps were performed in closed systems. Human studies were conducted in accordance with the Declaration of Helsinki and approved by the Institutional Review Board of Guy's Hospital (reference 09/H0707/86).

Expansion

In the BRC GMP Facility, cells were seeded in MACS GMP Cell Differentiation/Expansion Bags at 0.5×10^6 cells/mL in TexMACS GMP Medium (Miltenyi Biotec, Germany), supplemented with 5% human serum containing 100 nM rapamycin (Rapamune) and activated with anti-CD3- and anti-CD28-coated beads (4:1 bead:cell ratio, MACS GMP ExpAct Treg Kit, Miltenyi Biotec, Germany). Human recombinant IL-2 (500 IU/mL; Proleukin) was added at day 4–6 and replenished every 2 to 3 days. The cells were rested for 4 days before restimulation. Stimulation occurred on days 12 and 24, during which time cells were pooled, fresh beads (1:1), rapamycin and IL-2 were added, and the suspension was re-seeded at 0.5×10^6 cells/mL into new bags (250, 500, or 1,000 mL) For a schematic representation of the process see Figure 5. Expanded cells were harvested on day 36 and pooled. The ExpAct Treg expansion beads were depleted using the CliniMACS Plus System (Miltenyi Biotec) to form a bead-depleted cell population. A small aliquot of the cells was then taken for safety and functional analysis.

Cryopreservation

Because the ONE study is a multi-site trial, cryopreservation of the Tregs is required to accommodate for the transfer of these cells to different trial sites. In addition, the cryopreservation of Tregs allows the storage and dosing of patients to occur at specified dates in relation to transplantation (5 days after transplantation in the ONE study) and can prevent any issues that may occur due to unforeseen circumstances, which could alter transplantion dates.

After final harvest, all batches had to fulfill the set release criteria that included (1) $CD4^+CD25^+FOXP3^+$ cells ≥60% of the live cell population; (2) $CD8^+$ cells ≤10%; (3) ≤100 beads per 3×10^6 cells; (4) viability ≥70%; (5) sterility: no growth after 5 days (BacT/ALERT, Biomerieux); (6) endotoxin ≤175 IU/mL; (7) mycoplasma: not detected; and (8) suppression ≥60%. For cryopreservation, cells were pelleted by centrifugation and resuspended in CryoStor CS10 freezing media at a concentration to provide the dose of cells required in 2 mL. The product was transferred into CellSeal Cryovials, 2.1 mL per vial, and cooled to −80°C in a controlled rate freezer before transfer to liquid nitrogen (vapor phase) for long-term storage.

In order to assess the stability of the cryopreserved product and the effect of cryopreservation on the biology and function of the final product, cells were thawed rapidly and diluted in 5% human serum albumin, and the viability, recovery, phenotype, and suppressive function of the cryopreserved product were assessed.

Statistical Analysis

Statistical analysis was carried out on GraphPad Prism 5.0c (GraphPad software, CA, USA). Parametric and nonparametric data were expressed as mean ± standard error and median, where appropriate. For comparison of parametric data, paired and unpaired Student's t tests (for two samples) and 1-way ANOVA (for multiple comparisons) were carried out as appropriate. Statistical significance was set at $p < 0.05$. *$p < 0.05$, ** $p < 0.01$, and *** $p < 0.001$.

SUPPLEMENTAL INFORMATION

Supplemental Information includes one figure and one table and can be found with this article online at https://doi.org/10.1016/j.omtm.2018.01.006.

AUTHOR CONTRIBUTIONS

N.S. and H.F. contributed equally to this work. H.F., collection and assembly of data, data analysis, and interpretation; N.S., data collection and data analysis, writing of the manuscript, and critical revision of the manuscript; N.G. and C.S., data analysis and critical revision of the manuscript; S.T., K.L., L.J.F., A.H., and C.F., data collection and analysis in the GMP facility and critical revision of the manuscript; R.H., D.G., A.B., K.W., and R.L., providing samples from patients and critical revision of the article; G.L., design of the study, writing the manuscript, and providing funding.

CONFLICTS OF INTEREST

The authors declare no competing financial interest.

ACKNOWLEDGMENTS

This work was supported by the King's Health Partners Research and Development Challenge Fund, Guy's and St Thomas' Charity (grant no. R1405170), the British Heart Foundation, ONE Study, European Cooperation in Science and Technology (COST; part of the EU Framework Programme Horizon 2020) for the AFACTT project (Action to Focus and Accelerate Cell-based Tolerance inducing Therapies; BM1305), the Medical Research Council (MRC) (within the MRC Centre for Transplantation, King's College London, UK; MRC grant no. MR/J006742/1), and the National Institute for Health Research (NIHR) Biomedical Research Centre at Guy's and St Thomas' NHS Foundation Trust and King's College London. The views expressed are those of the author(s) and not necessarily those of the NHS, the NIHR, or the Department of Health.

REFERENCES

1. Chatenoud, L., and Bach, J.F. (2005). Regulatory T cells in the control of autoimmune diabetes: the case of the NOD mouse. Int. Rev. Immunol. *24*, 247–267.
2. Kohm, A.P., Carpentier, P.A., Anger, H.A., and Miller, S.D. (2002). Cutting edge: CD4+CD25+ regulatory T cells suppress antigen-specific autoreactive immune responses and central nervous system inflammation during active experimental autoimmune encephalomyelitis. J. Immunol. *169*, 4712–4716.
3. Morgan, M.E., Flierman, R., van Duivenvoorde, L.M., Witteveen, H.J., van Ewijk, W., van Laar, J.M., de Vries, R.R., and Toes, R.E. (2005). Effective treatment of collagen-induced arthritis by adoptive transfer of CD25+ regulatory T cells. Arthritis Rheum. *52*, 2212–2221.
4. Lindley, S., Dayan, C.M., Bishop, A., Roep, B.O., Peakman, M., and Tree, T.I. (2005). Defective suppressor function in CD4(+)CD25(+) T-cells from patients with type 1 diabetes. Diabetes *54*, 92–99.
5. van Amelsfort, J.M., Jacobs, K.M., Bijlsma, J.W., Lafeber, F.P., and Taams, L.S. (2004). CD4(+)CD25(+) regulatory T cells in rheumatoid arthritis: differences in the presence, phenotype, and function between peripheral blood and synovial fluid. Arthritis Rheum. *50*, 2775–2785.
6. Tsang, J.Y., Tanriver, Y., Jiang, S., Leung, E., Ratnasothy, K., Lombardi, G., and Lechler, R. (2009). Indefinite mouse heart allograft survival in recipient treated with CD4(+)CD25(+) regulatory T cells with indirect allospecificity and short term immunosuppression. Transpl. Immunol. *21*, 203–209.
7. Golshayan, D., Jiang, S., Tsang, J., Garin, M.I., Mottet, C., and Lechler, R.I. (2007). In vitro-expanded donor alloantigen-specific CD4+CD25+ regulatory T cells promote experimental transplantation tolerance. Blood *109*, 827–835.
8. Joffre, O., Santolaria, T., Calise, D., Al Saati, T., Hudrisier, D., Romagnoli, P., and van Meerwijk, J.P. (2008). Prevention of acute and chronic allograft rejection with CD4+CD25+Foxp3+ regulatory T lymphocytes. Nat. Med. *14*, 88–92.
9. Feng, G., Nadig, S.N., Bäckdahl, L., Beck, S., Francis, R.S., Schiopu, A., Whatcott, A., Wood, K.J., and Bushell, A. (2011). Functional regulatory T cells produced by inhibiting cyclic nucleotide phosphodiesterase type 3 prevent allograft rejection. Sci. Transl. Med. *3*, 83ra40.
10. Taylor, P.A., Panoskaltsis-Mortari, A., Swedin, J.M., Lucas, P.J., Gress, R.E., Levine, B.L., June, C.H., Serody, J.S., and Blazar, B.R. (2004). L-selectin(hi) but not the L-selectin(lo) CD4+25+ T-regulatory cells are potent inhibitors of GVHD and BM graft rejection. Blood *104*, 3804–3812.
11. Taylor, P.A., Lees, C.J., and Blazar, B.R. (2002). The infusion of ex vivo activated and expanded CD4(+)CD25(+) immune regulatory cells inhibits graft-versus-host disease lethality. Blood *99*, 3493–3499.
12. Putnam, A.L., Safinia, N., Medvec, A., Laszkowska, M., Wray, M., Mintz, M.A., Trotta, E., Szot, G.L., Liu, W., Lares, A., et al. (2013). Clinical grade manufacturing of human alloantigen-reactive regulatory T cells for use in transplantation. Am. J. Transplant. *13*, 3010–3020.
13. Sagoo, P., Ali, N., Garg, G., Nestle, F.O., Lechler, R.I., and Lombardi, G. (2011). Human regulatory T cells with alloantigen specificity are more potent inhibitors of alloimmune skin graft damage than polyclonal regulatory T cells. Sci. Transl. Med. *3*, 83ra42.
14. Xiao, F., Ma, L., Zhao, M., Huang, G., Mirenda, V., Dorling, A., Lechler, R., and Lombardi, G. (2014). Ex vivo expanded human regulatory T cells delay islet allograft rejection via inhibiting islet-derived monocyte chemoattractant protein-1 production in CD34+ stem cells-reconstituted NOD-scid IL2rγnull mice. PLoS One *9*, e90387.
15. Nadig, S.N., Wieckiewicz, J., Wu, D.C., Warnecke, G., Zhang, W., Luo, S., Schiopu, A., Taggart, D.P., and Wood, K.J. (2010). In vivo prevention of transplant arteriosclerosis by ex vivo-expanded human regulatory T cells. Nat. Med. *16*, 809–813.
16. Issa, F., Hester, J., Goto, R., Nadig, S.N., Goodacre, T.E., and Wood, K. (2010). Ex vivo-expanded human regulatory T cells prevent the rejection of skin allografts in a humanized mouse model. Transplantation *90*, 1321–1327.
17. Wu, D.C., Hester, J., Nadig, S.N., Zhang, W., Trzonkowski, P., Gray, D., Hughes, S., Johnson, P., and Wood, K.J. (2013). Ex vivo expanded human regulatory T cells can prolong survival of a human islet allograft in a humanized mouse model. Transplantation *96*, 707–716.
18. Brunstein, C.G., Miller, J.S., Cao, Q., McKenna, D.H., Hippen, K.L., Curtsinger, J., Defor, T., Levine, B.L., June, C.H., Rubinstein, P., et al. (2011). Infusion of ex vivo expanded T regulatory cells in adults transplanted with umbilical cord blood: safety profile and detection kinetics. Blood *117*, 1061–1070.

19. Di Ianni, M., Falzetti, F., Carotti, A., Terenzi, A., Castellino, F., Bonifacio, E., Del Papa, B., Zei, T., Ostini, R.I., Cecchini, D., et al. (2011). Tregs prevent GVHD and promote immune reconstitution in HLA-haploidentical transplantation. Blood *117*, 3921–3928.

20. Marek-Trzonkowska, N., Myśliwiec, M., Dobyszuk, A., Grabowska, M., Derkowska, I., Juścińska, J., Owczuk, R., Szadkowska, A., Witkowski, P., Młynarski, W., et al. (2014). Therapy of type 1 diabetes with CD4(+)CD25(high)CD127-regulatory T cells prolongs survival of pancreatic islets - results of one year follow-up. Clin. Immunol. *153*, 23–30.

21. Trzonkowski, P., Bieniaszewska, M., Juścińska, J., Dobyszuk, A., Krzystyniak, A., Marek, N., Myśliwska, J., and Hellmann, A. (2009). First-in-man clinical results of the treatment of patients with graft versus host disease with human ex vivo expanded CD4+CD25+CD127- T regulatory cells. Clin. Immunol. *133*, 22–26.

22. Strauss, L., Whiteside, T.L., Knights, A., Bergmann, C., Knuth, A., and Zippelius, A. (2007). Selective survival of naturally occurring human CD4+CD25+Foxp3+ regulatory T cells cultured with rapamycin. J. Immunol. *178*, 320–329.

23. Battaglia, M., Stabilini, A., and Roncarolo, M.G. (2005). Rapamycin selectively expands CD4+CD25+FoxP3+ regulatory T cells. Blood *105*, 4743–4748.

24. Scottà, C., Esposito, M., Fazekasova, H., Fanelli, G., Edozie, F.C., Ali, N., Xiao, F., Peakman, M., Afzali, B., Sagoo, P., et al. (2013). Differential effects of rapamycin and retinoic acid on expansion, stability and suppressive qualities of human CD4(+)CD25(+)FOXP3(+) T regulatory cell subpopulations. Haematologica *98*, 1291–1299.

25. Araki, K., Turner, A.P., Shaffer, V.O., Gangappa, S., Keller, S.A., Bachmann, M.F., Larsen, C.P., and Ahmed, R. (2009). mTOR regulates memory CD8 T-cell differentiation. Nature *460*, 108–112.

26. Golovina, T.N., Mikheeva, T., Brusko, T.M., Blazar, B.R., Bluestone, J.A., and Riley, J.L. (2011). Retinoic acid and rapamycin differentially affect and synergistically promote the ex vivo expansion of natural human T regulatory cells. PLoS One *6*, e15868.

27. Koenen, H.J., Fasse, E., and Joosten, I. (2005). CD27/CFSE-based ex vivo selection of highly suppressive alloantigen-specific human regulatory T cells. J. Immunol. *174*, 7573–7583.

28. Koenen, H.J., Smeets, R.L., Vink, P.M., van Rijssen, E., Boots, A.M., and Joosten, I. (2008). Human CD25highFoxp3pos regulatory T cells differentiate into IL-17-producing cells. Blood *112*, 2340–2352.

29. Fletcher, J.M., Lonergan, R., Costelloe, L., Kinsella, K., Moran, B., O'Farrelly, C., Tubridy, N., and Mills, K.H. (2009). CD39+Foxp3+ regulatory T Cells suppress pathogenic Th17 cells and are impaired in multiple sclerosis. J. Immunol. *183*, 7602–7610.

30. Hori, S. (2010). Developmental plasticity of Foxp3+ regulatory T cells. Curr. Opin. Immunol. *22*, 575–582.

31. Afzali, B., Edozie, F.C., Fazekasova, H., Scottà, C., Mitchell, P.J., Canavan, J.B., Kordasti, S.Y., Chana, P.S., Ellis, R., Lord, G.M., et al. (2013). Comparison of regulatory T cells in hemodialysis patients and healthy controls: implications for cell therapy in transplantation. Clin. J. Am. Soc. Nephrol. *8*, 1396–1405.

32. Peters, J.H., Hilbrands, L.B., Koenen, H.J., and Joosten, I. (2008). Ex vivo generation of human alloantigen-specific regulatory T cells from CD4(pos)CD25(high) T cells for immunotherapy. PLoS One *3*, e2233.

33. Kreher, C.R., Dittrich, M.T., Guerkov, R., Boehm, B.O., and Tary-Lehmann, M. (2003). CD4+ and CD8+ cells in cryopreserved human PBMC maintain full functionality in cytokine ELISPOT assays. J. Immunol. Methods *278*, 79–93.

34. Weinberg, A., Song, L.Y., Wilkening, C., Sevin, A., Blais, B., Louzao, R., Stein, D., Defechereux, P., Durand, D., Riedel, E., et al.; Pediatric ACTG Cryopreservation Working Group (2009). Optimization and limitations of use of cryopreserved peripheral blood mononuclear cells for functional and phenotypic T-cell characterization. Clin. Vaccine Immunol. *16*, 1176–1186.

35. Brooks-Worrell, B., Tree, T., Mannering, S.I., Durinovic-Bello, I., James, E., Gottlieb, P., Wong, S., Zhou, Z., Yang, L., Cilio, C.M., et al.; T-Cell Workshop Committee, Immunology of Diabetes Society (2011). Comparison of cryopreservation methods on T-cell responses to islet and control antigens from type 1 diabetic patients and controls. Diabetes Metab. Res. Rev. *27*, 737–745.

36. Mallone, R., Mannering, S.I., Brooks-Worrell, B.M., Durinovic-Belló, I., Cilio, C.M., Wong, F.S., and Schloot, N.C.; T-Cell Workshop Committee, Immunology of Diabetes Society (2011). Isolation and preservation of peripheral blood mononuclear cells for analysis of islet antigen-reactive T cell responses: position statement of the T-Cell Workshop Committee of the Immunology of Diabetes Society. Clin. Exp. Immunol. *163*, 33–49.

37. Sasnoor, L.M., Kale, V.P., and Limaye, L.S. (2003). Supplementation of conventional freezing medium with a combination of catalase and trehalose results in better protection of surface molecules and functionality of hematopoietic cells. J. Hematother. Stem Cell Res. *12*, 553–564.

38. Basu, S., Golovina, T., Mikheeva, T., June, C.H., and Riley, J.L. (2008). Cutting edge: Foxp3-mediated induction of pim 2 allows human T regulatory cells to preferentially expand in rapamycin. J. Immunol. *180*, 5794–5798.

39. Zeiser, R., Leveson-Gower, D.B., Zambricki, E.A., Kambham, N., Beilhack, A., Loh, J., Hou, J.Z., and Negrin, R.S. (2008). Differential impact of mammalian target of rapamycin inhibition on CD4+CD25+Foxp3+ regulatory T cells compared with conventional CD4+ T cells. Blood *111*, 453–462.

40. Delgoffe, G.M., Kole, T.P., Zheng, Y., Zarek, P.E., Matthews, K.L., Xiao, B., Worley, P.F., Kozma, S.C., and Powell, J.D. (2009). The mTOR kinase differentially regulates effector and regulatory T cell lineage commitment. Immunity *30*, 832–844.

41. Hester, J., Schiopu, A., Nadig, S.N., and Wood, K.J. (2012). Low-dose rapamycin treatment increases the ability of human regulatory T cells to inhibit transplant arteriosclerosis in vivo. Am. J. Transplant. *12*, 2008–2016.

42. Hoffmann, P., Boeld, T.J., Eder, R., Huehn, J., Floess, S., Wieczorek, G., Olek, S., Dietmaier, W., Andreesen, R., and Edinger, M. (2009). Loss of FOXP3 expression in natural human CD4+CD25+ regulatory T cells upon repetitive in vitro stimulation. Eur. J. Immunol. *39*, 1088–1097.

43. Hippen, K.L., Merkel, S.C., Schirm, D.K., Sieben, C.M., Sumstad, D., Kadidlo, D.M., McKenna, D.H., Bromberg, J.S., Levine, B.L., Riley, J.L., et al. (2011). Massive ex vivo expansion of human natural regulatory T cells (T(regs)) with minimal loss of in vivo functional activity. Sci. Transl. Med. *3*, 83ra41.

44. Cosmi, L., Maggi, L., Santarlasci, V., Capone, M., Cardilicchia, E., Frosali, F., Querci, V., Angeli, R., Matucci, A., Fambrini, M., et al. (2010). Identification of a novel subset of human circulating memory CD4(+) T cells that produce both IL-17A and IL-4. J. Allergy Clin Immunol. *125*, 222–230, e1-4.

45. Tresoldi, E., Dell'Albani, I., Stabilini, A., Jofra, T., Valle, A., Gagliani, N., Bondanza, A., Roncarolo, M.G., and Battaglia, M. (2011). Stability of human rapamycin-expanded CD4+CD25+ T regulatory cells. Haematologica *96*, 1357–1365.

46. King, M.A., Covassin, L., Brehm, M.A., Racki, W., Pearson, T., Leif, J., Laning, J., Fodor, W., Foreman, O., Burzenski, L., et al. (2009). Human peripheral blood leucocyte non-obese diabetic-severe combined immunodeficiency interleukin-2 receptor gamma chain gene mouse model of xenogeneic graft-versus-host-like disease and the role of host major histocompatibility complex. Clin. Exp. Immunol. *157*, 104–118.

OMTM, Volume *8*

Supplemental Information

A Rapamycin-Based GMP-Compatible Process for the Isolation and Expansion of Regulatory T Cells for Clinical Trials

Henrieta Fraser, Niloufar Safinia, Nathali Grageda, Sarah Thirkell, Katie Lowe, Laura J. Fry, Cristiano Scottá, Andrew Hope, Christopher Fisher, Rachel Hilton, David Game, Paul Harden, Andrew Bushell, Kathryn Wood, Robert I. Lechler, and Giovanna Lombardi

Supplementary Material

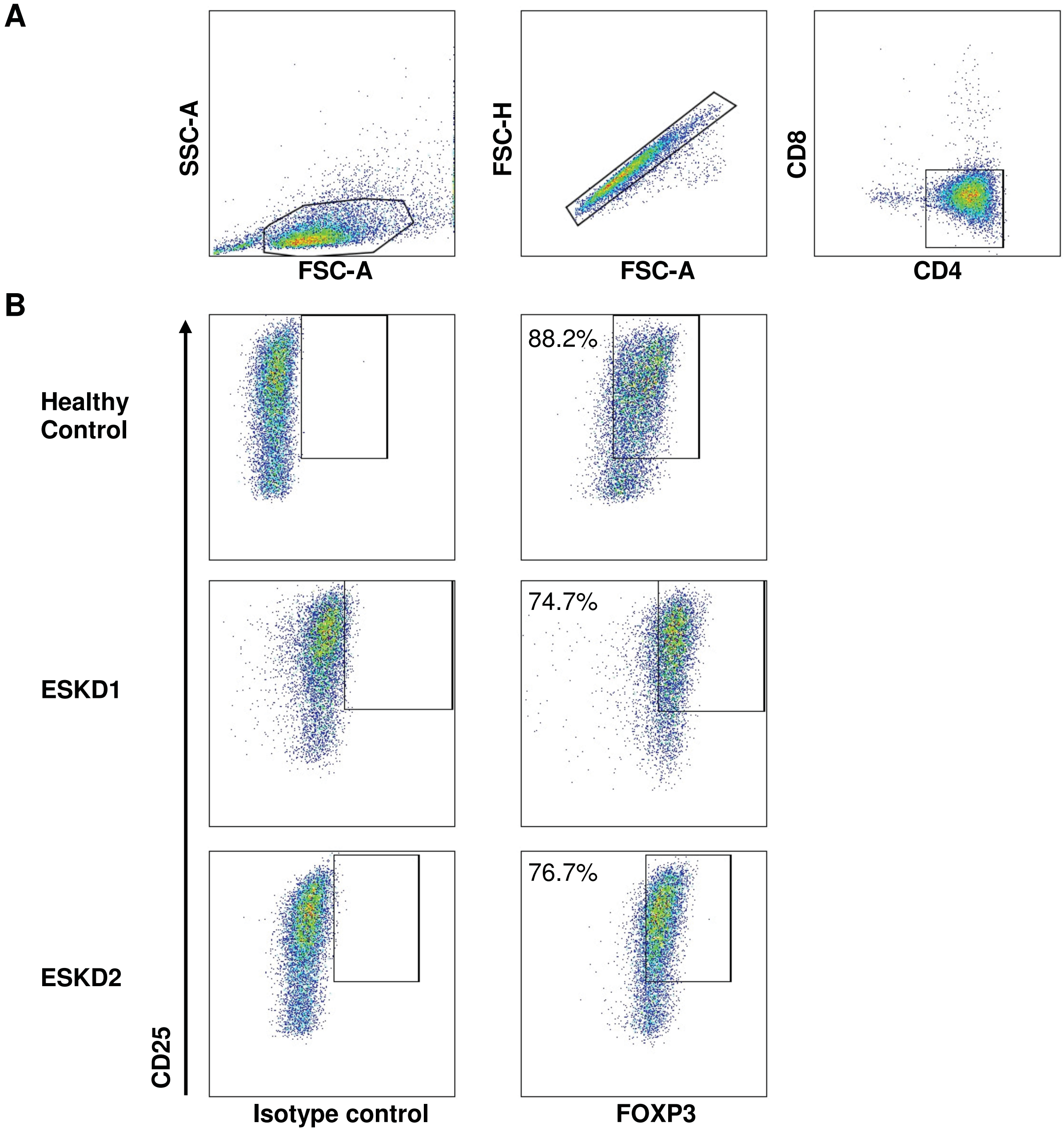

Figure S1: Gating strategy and flow cytometric staining patterns. A. Gating strategy for identification of $CD4^+CD25^+FOXP3^+$ cells. Cell doublets were excluded from the analysis on the basis of forward scatter area and height (FSC-A and FSC-H, respectively). **B.** Dot plots showing the percentage of $CD4^+CD25^+FOXP3^+$ cells for one healthy control and two patients included in the study.

Table S1: Demographics of patients included in the study.

	Gender	Ethnicity	Age	Therapeutic agents	Condition leading to ESKD (Months on dialysis prior recruitment)	Co-morbidities
**ESKD1**	Female	Caucasian	52	Venofer on dialysis<br><br>Adcal<br>Candesartan<br>Alfacalcidol	Autosomal dominant polycystic kidney disease (50)	Obesity<br>(gastric band 2011)
**ESKD2**	Female	Afro-Caribbean	49	Epo, iron and alfacalcidol on dialysis<br><br>Atorvastatin<br>Atenolol<br>Amlodipine<br>Candesartan<br>Omeprazole<br>Tramadol	Hypertension (106)	Hyperparathyroidism /parathyroidectomy 2009<br>Ischaemic heart disease
